# First molecular detection and characterization of Marek’s disease virus in red-crowned cranes (*Grus japonensis*): a case report

**DOI:** 10.1186/s12917-018-1437-9

**Published:** 2018-04-03

**Authors:** Xue Lian, Xin Ming, Jiarong Xu, Wangkun Cheng, Xunhai Zhang, Hongjun Chen, Chan Ding, Yong-Sam Jung, Yingjuan Qian

**Affiliations:** 1 MOE Joint International Research Laboratory of Animal Health and Food Safety, College of Veterinary Medicine, Nanjing Agricultural University, Nanjing, China; 2 Nanjing Hongshan Forest Zoo, Nanjing, China; 3 Anhui Provincial Key Laboratory for Control and Monitoring of Poultry Diseases, Anhui Science and Technology University, Fengyang, China; 4 Shanghai Veterinary Research Institute, Chinese Academy of Agricultural Sciences, Shanghai, China

**Keywords:** Marek’s disease virus, Red-crowned crane, Clinical necropsy, PCR, Homology

## Abstract

**Background:**

Marek’s disease virus (MDV) resides in the genus *Mardivirus* in the family *Herpesviridae*. MDV is a highly contagious virus that can cause neurological lesions, lymphocytic proliferation, immune suppression, and death in avian species, including Galliformes (chickens, quails, partridges, and pheasants), Strigiformes (owls), Anseriformes (ducks, geese, and swans), and Falconiformes (kestrels).

**Case presentation:**

In 2015, two red-crowned cranes died in Nanjing (Jiangsu, China). It was determined that the birds were infected with Marek’s disease virus by histopathological examination, polymerase chain reaction (PCR), gene sequencing and sequence analysis of tissue samples from two cranes. Gross lesions included diffuse nodules in the skin, muscle, liver, spleen, kidney, gizzard and heart, along with liver enlargement and gizzard mucosa hemorrhage. Histopathological assay showed that infiltrative lymphocytes and mitotic figures existed in liver and heart. The presence of MDV was confirmed by PCR. The sequence analysis of the Meq gene showed 100% identity with Md5, while the VP22 gene showed the highest homology with CVI988. Furthermore, the phylogenetic analysis of the VP22 and Meq genes suggested that the MDV (from cranes) belongs to MDV serotype 1.

**Conclusion:**

We describe the first molecular detection of Marek’s disease in red-crowned cranes based on the findings previously described. To our knowledge, this is also the first molecular identification of Marek’s disease virus in the order Gruiformes and represents detection of a novel MDV strain.

## Background

The red-crowned crane (*Grus japonensis*) is classified as an endangered species with a small global population of 1830 mature individuals [1]. The red-crowned crane breeds in south-eastern Russia, north-east China, Mongolia, and eastern Hokkaido, Japan [2]. The Russian and Chinese populations mainly migrate to the Yellow River Delta and the coast of Jiangsu province, China, and the demilitarized zone of North Korea/South Korea in winter [3]. The number of over wintering cranes in China is now only 8% of what it was in the 1980s due to habitat degradation [4]. This also leads to the overconcentration of cranes at a few sites, which therefore decrease the genetic diversity [5]. The small population and low genetic diversity make this species especially vulnerable to epidemic diseases.

Marek’s disease (MD) is a highly contagious disease, characterized by immunosuppression, neurological disorder, CD4^+^ T cells transformation and eventual tumor formation in peripheral nerves and visceral organs [6]. The causative agent of MD is Marek’s disease virus (MDV) which is a member of the genus *Mardivirus* belonging to the subfamily *Alphaherpesvirinae* of the family *Herpesviridae.* MD was first reported in chicken by Josef Marek in 1907 [7], and is prevalent throughout the world. Later, it was also reported in Galliformes (such as turkeys [8], quails [9] and pheasants [10]), Strigiformes (owls), Anseriformes (ducks, geese and swans) and Falconiformes (kestrels). The most important natural hosts for MDV are domestic and wild chicken, including game fowl [11], native breeds [12] and jungle fowl [13]. MDV contains three serotypes: MDV-1 (GaHV-2), MDV-2 (GaHV-3) and MDV-3 (MeHV-1) [14]. However, only MDV-1 can induce disease in chicken, whereas MDV-2 and MDV-3 are avirulent and are used as vaccines. Symptoms of MD depend on the age of the bird [15, 16]. For example, young chickens infected with virulent MDV strains may exhibit high mortality in 8–16 days post infection with early mortality syndrome [17]. MDV can be transmitted horizontally through aerosols and enter the host through the respiratory tract, but it cannot be transmitted vertically from chicken to eggs [18]. The infectious virus particles replicate in epithelial cells of the feather follicles, and then are shed to the environment with the skin dander [19]. MDV in the dust survives for at least several months at room temperature [20]. Thus, skin dander, house dust, feces, and saliva can be a source of infection. Because cross-species spreading of the virus is possible, this mode of transmission enables it to infect an even broader spectrum of hosts [21]. Despite several cases of herpesvirus infection in cranes that were previously reported [22, 23], there is a lack of molecular evidence on MDV infection and knowledge of which species are infected.

This article reports the molecular detection of a novel MDV isolate in the red-crowned crane. It is also the first molecular determination of MDV from individual red-crowned cranes (*Grus japonensis*), indicating that red-crowned cranes can be infected by MDV.

## Case presentation

In August 2015, reduced feed consumption was observed in a 2-month-old red-crowned crane (crane A) that was bred and incubated in Hongshan Forest Zoo in Nanjing, China. After segregation and palliative treatment, it exhibited astasia and then died. Subcutaneous palpation examination revealed many diffuse soybean-sized nodules (Table 1). Red-crowned crane B showed obvious swelling of its left knee joint. After antibiotic treatment, although it showed an improved appetite and a decrease in symptoms, the crane died unexpectedly ten days later (Table 1). The dead cranes were not immunized with the MDV vaccine. Although there had been no previous reports of MDV-infected cranes, the cranes were fed in mesh cages that were accessible to wild birds.

**Table 1:** Timeline table of the information in the case report

Date	Clinical Observation	Intervention	Samples Collected
Aug 19-20	Red-crowned crane A^a^: emaciation, reduced appetite. Diagnostic check: Heart rate: 106 bp/min Body temperature: 39.7°C Weight: 1.6kg; Crane A^a^: astasia.	Rocephin (100 mg/kg) + Dexamethasone (0.25 mg/kg) + 0.9% NaCl 15 ml (intravenous infusion once daily for 3 days) Vitamin C (100 mg/kg) + Vitamin B_1_(15 mg/kg) + ATP (5 mg/kg) + Inosine (15 mg/kg) + coenzyme A (15 IU/kg) + 10% glucose 15 ml (intravenous infusion once daily for 3 days) 10% glucose + mixture of fish and corn, orally, for 3 days Calcium gluconate (25 mg/kg) + 10% glucose 15 ml (intravenous infusion once daily), for 1 day	Crane A^a^: •Histopathological examination of liver, heart, and spleen. Three repeated samples were collected from each tissue. The ratio of MDV-positive tissues: Heart: 67%, liver: 100%, spleen: 67%. •RNA extraction of feather, liver, spleen, kidney and muscle. Three of each. Positive ratio of all samples was 100%. •Genome extraction of muscle, feather, feather-pulp and spleen; three of each. The positive ratio of all samples was 100%. Crane B^b^: same as crane A.
Aug 21	Diagnostic check: Heart rate: 156 bp/min Body temperature: 39.8°C Crane A^a^ died.		
Aug 22	Clinical necropsy: Nodules found in skin, muscle, liver, heart, trachea, and gizzard. (MD suspected).		
Sept 11	Crane B^b^: obvious swelling of its left knee joint.	Musk analgesic aerosol, external use	
Sept 12-17	Clinical improvement: Recovered appetite, left knee joint swelled	Musk analgesic aerosol + ichthammol ointment, external use Calcium tablets (10 mg/kg) + Vitamin D_2_(500 IU/kg) + Vitamin A (1500 IU/kg), orally	
Sept 18-19	Relapse, anorexia, depression		
Sept 20	Crane B^b^ died. Clinical necropsy: Inflammatory exudates; diffuse yellow-white nodules in muscle, liver, spleen, kidney, pancreas, gizzard, and heart.		

^a^Crane A: hatched on June 10, died on Aug 22, 2015. Body weight: 1.6 kg

^b^Crane B: hatched on June 9, died on Sep 22. Body weight: 2.4 kg

**Fig. 1:**
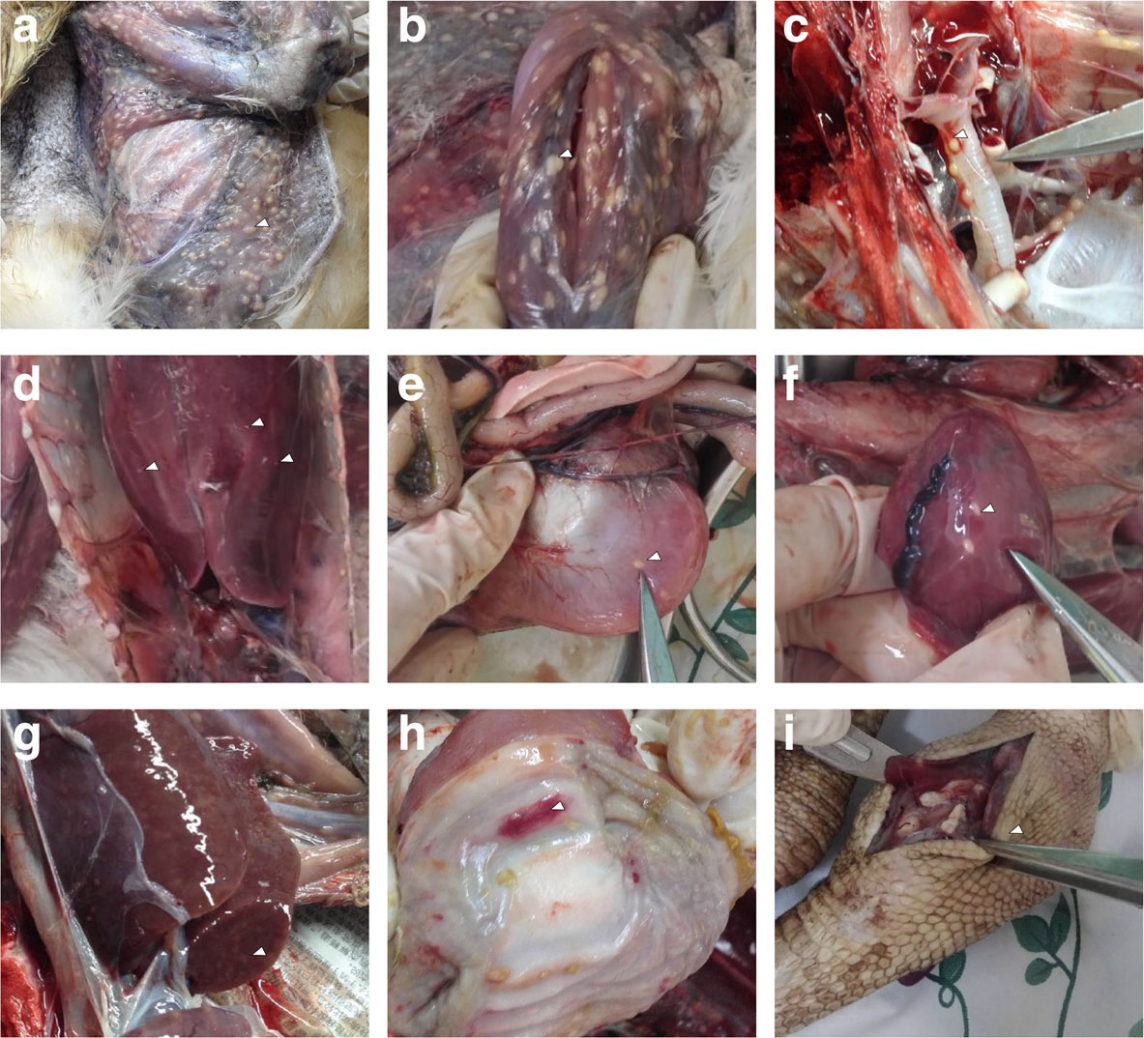
Clinical symptoms and pathological lesions. **a**-**f**, Nodules in the (**a**) skin, (**b**) muscle, (**c**) trachea, (**d**) liver, (**e**) gizzard, (**f**) heart; **g**, nodules in the vertical section of the liver; **h**, hemorrhage sites in the gizzard mucosa; **i**, left knee joint swelling

Clinical necropsy revealed diffuse yellow-white nodules from millet to soybean size in the skin, muscle, trachea, liver, gizzard, and heart in both cranes (Fig. 1a-f). Diffused yellow-white nodules were also observed in the vertical section of the liver (Fig. 1g). Several hemorrhage sites were observed in the gizzard mucosa (Fig. 1h). A large amount of inflammatory exudates oozed from a surface cut of the swollen joint (Fig. 1i). No other significant gross lesions were observed.

**Fig. 2:**
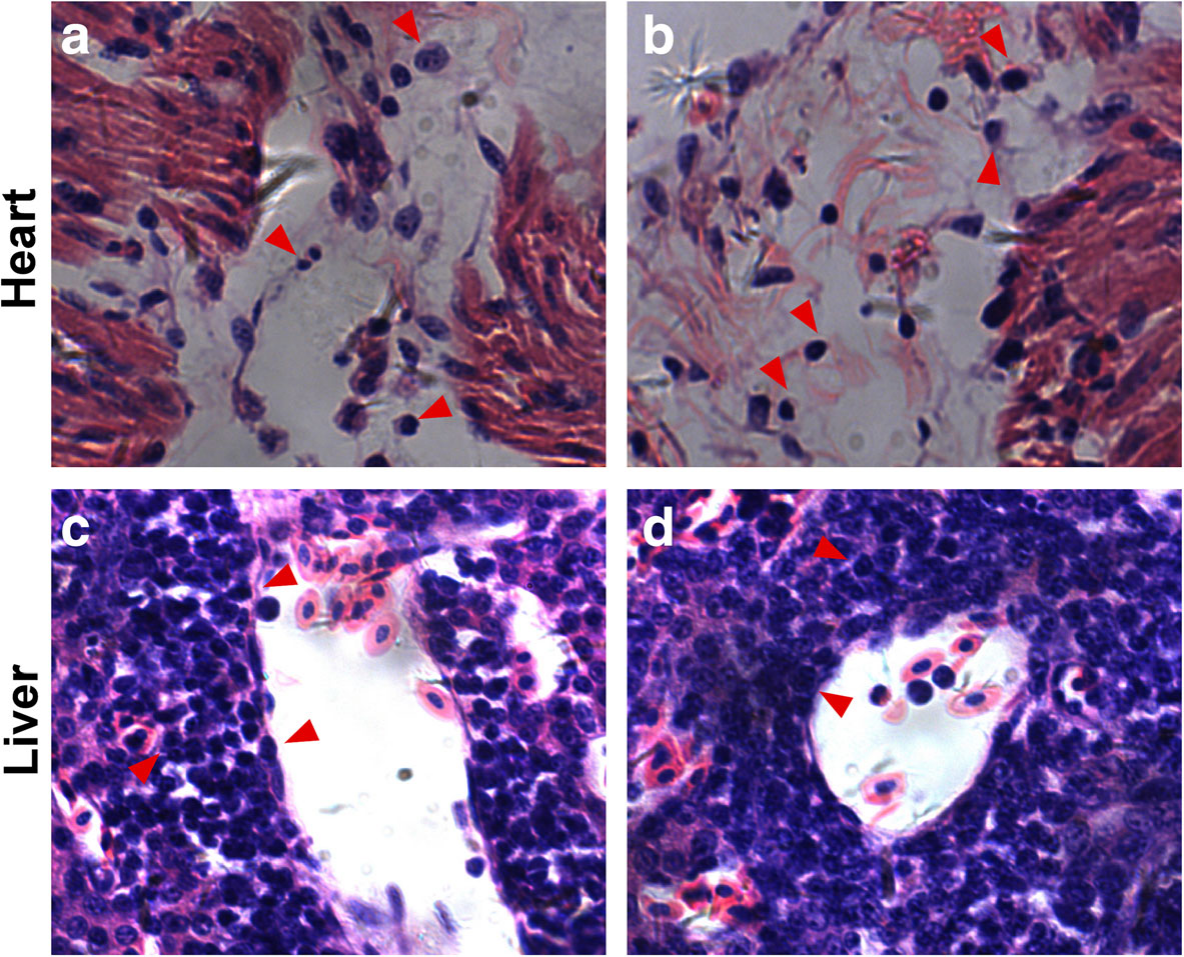
Histopathological section (H&E staining) of the liver and heart. **a** and **b**, lymphomatous infiltration in the liver; **c** and **d**, lymphomatous infiltration in the heart. The arrow shows a range of leukocytes, including large lymphocytes, small lymphocytes lymphoblasts, and malignant cells with mitotic figure.

Representative tissue samples were collected during necropsy from the liver, spleen, kidney, muscle, heart, feather follicles, and skin, and these were used for pathological and virological investigations (Table 1). Two sets of tissue samples were collected for different purposes, and were processed in different ways: one set was used for virology analysis and stored at − 80 °C and the other was used for conventional histopathology analysis and fixed in 10% neutral formalin. Formalin-fixed samples were subsequently dehydrated through graded alcohols before being embedded in paraffin wax. Several 4 μm-thick sections were cut from each sample and stained using hematoxylin and eosin (H&E). The heart histology showed that numerous lymphoid cells had infiltrated between adjacent cardiac muscle fibers. We also found that there were various polymorphic lymphoid cells with mitotic figures (Fig. 2a-b). Similarly, the liver structure was disrupted, and a large number of variably sized lymphoblastic cells was dispersed or exhibited mass distribution in the hepatic lobules (Fig. 2c-d). These findings were consistent with the symptoms of MDV infection. However, bacterial infection cannot be excluded from this case, as swollen joints could be a consequence of bacterial infection. It is possible that the birds were infected with MDV, which is immunosuppressive, but succumbed to other conditions such as bacterial superinfection.

**Fig. 3:**
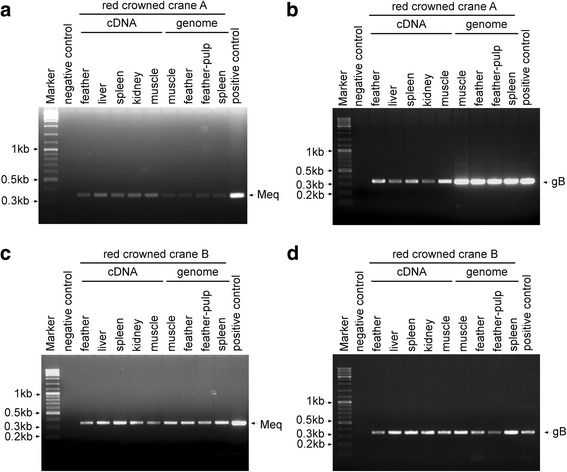
Agarose gel electrophoresis of MDV from RT-PCR of the Meq and gB genes. **a** and **c**, Meq (347 bp, partial) was amplified from cDNAs and genomes of different tissues. **b** and **d**, gB (338 bp, partial) was amplified from cDNAs and genomes of different tissues. The feather sample was a homogenate of ground feathers and skin that contained feather follicle epithelium. The entire cell genome from Md5-infected CEF cells was used as a positive control. The cDNA from CEF cells was used as a negative control

For virological detection, frozen tissue samples of feather, liver, spleen, kidney, and muscle were homogenized using a dispersing homogenizer (IKA Ultra-Turrax, T10, Germany). Total RNA was extracted using TRIzol reagent (Sigma, USA) according to the manufacturer’s protocol. The cDNA was prepared using an iScript™ Reverse Transcription Supermix kit (Bio-Rad, USA). The PCR was carried out with 2× Taq Master Mix (Vazyme, China) with the following conditions: denaturation at 94 °C for 5 min; 28 cycles of denaturation at 94 °C for 30 s, annealing at 55 °C for 30 s, and extension at 72 °C for 30s; final extension at 72 °C for 7 min; and maintenance at 4 °C. To optimize the PCR conditions, primers were modified on the basis of Yamaguchi et al. [24]. The Meq primer used herein amplified the DNA 10 bp longer than published primer, due to the forward primer being located 10 bp upstream from the published forward primer. For example, the sequence of the published primer is 5’-GGTCGACTTCGAGACGGAAA-3′, while here, we used 5’-CTTTCTCTCGGGTCGACTTC-3′. The underlined parts are the overlapped sequence. Our gB primers can amplify shorter sequences, but still within the same region compared with the published one. The amplified fragments were resolved by electrophoresis in 1.5% agarose gels (Sigma, USA). Here, RT-PCR was used to determine the viral gene expression. Meq mRNA was examined to measure general mRNA expression regardless of the phase of infection, and gB mRNA was examined to indicate virus lytic replication. Samples were analyzed for the presence of MDV and the reactions were positive for all sampled tissues when amplified with both Meq (Fig. 3a) and gB (Fig. 3b) primers [24] in both crane A (Fig. 3a, b) and crane B (Fig. 3c, d).

**Fig. 4:**
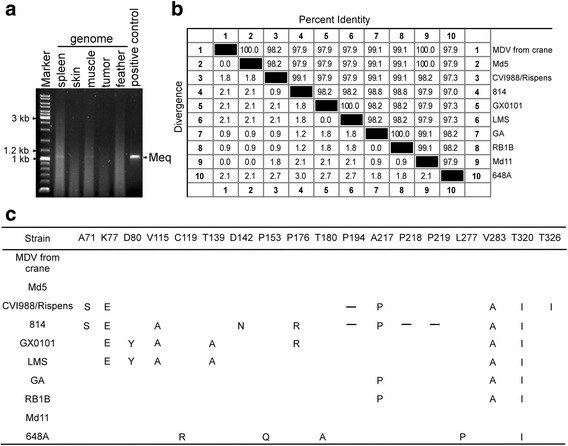
Amino acid sequence alignment of Meq (from crane). **a**, The full-length Meq gene (1020 bp) was amplified from the spleen genome. **b**, The amino acid (aa) sequences of Meq (339 aa) from cranes were aligned with 9 previously published MDV isolates (including CVI988, 814, GX0101, LMS, GA, RB-1B, Md11 and 648a). **c**, Substituted amino acids are listed, while deleted amino acids are denoted by strips in the alignment

**Fig. 5:**
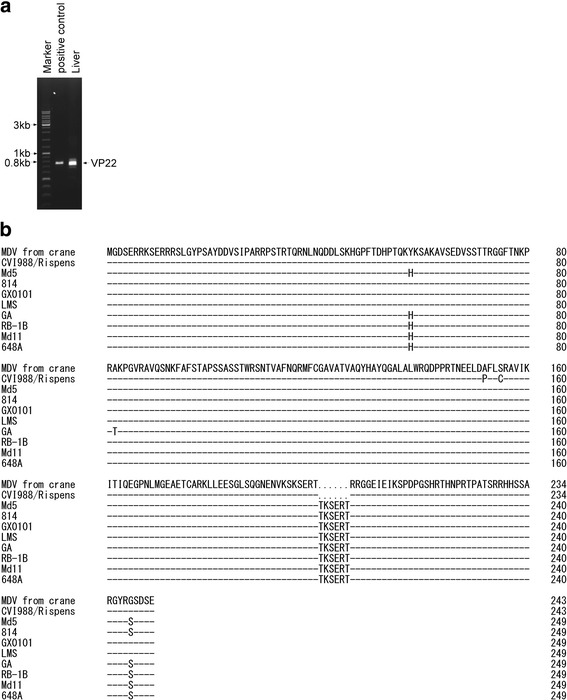
Amino acid sequence alignment of VP22 (from crane). **a**, The full-length VP22 gene (750 bp) was amplified from the liver genome. **b**, The amino acid sequence of VP22 (243 aa) from cranes was aligned with 9 previously published MDV isolates (including CVI988, 814, GX0101, LMS, GA, RB-1B, Md11 and 648a). Identical amino acids are denoted by strips in the alignment, and deleted amino acids are denoted by dots in the alignment

To further characterize the virus, samples from spleen, skin, muscle, feather and feather pulp were cut and digested overnight at 55 °C in lysis buffer (10 mM Tris, 100 mM EDTA, 0.5% SDS, and 0.2 mg/ml Proteinase K), and total cellular DNA was extracted with phenol-chloroform-isoamylalcohol (25:24:1), then precipitated with ethanol, and dissolved in TE buffer (10 mM Tris-HCl 1 mM EDTA pH 8.0). Total cellular DNA samples were used as templates for amplifying viral genes. The PCR was carried out with LA Taq DNA polymerase (Takara, Japan). Primers were designed to target the entire-length of the Meq and VP22 genes of Md5 (Table 2). The Meq gene was successfully amplified from the spleen tissue (Fig. 4a). The sequence alignment revealed that the analyzed fragment presented 100% sequence identity with reference MDV strains such as Md5 and Md11 (Fig. 4b and c). It also shared a high homology (97.9%) to the very virulent strain LMS from China (Fig. 4b). An additional PCR was carried out to amplify the VP22 gene, which is a major structural component of the virion [25] (Fig. 5a). The obtained band was sequenced and aligned using DNAMAN software. The sequence alignment showed that the VP22 protein shared the highest identity with the vaccine strain CVI988 as compared to other strains (Fig. 5b). Two substitutions at residues 152 (A → P), 155 (S → C) were found in CVI988, while 2–3 substitutions were found when aligned with other eight isolates. The same as CVI988, six amino acids (TKSERT) were deleted from residues 201 to 206 compared with other isolates (Fig. 5b). The deleted site was located in the domain (SKSERTTKSERT), which contained two copies of motif (KSERT) in virulent MDV strains. It has been shown that regardless of the deletion, the VP22 of the CVI988/Rispens vaccine strain still maintains an intercellular-trafficking function [26].

**Table 2:** Primers used for amplification of MDV viral genes

Name	Sequence	Length
Meq-RT-F	CTTTCTCTCGGGTCGACTTC	347 bp
Meq-RT-R	GTAAGCAGTCCAAGGGTCAC	
gB-RT-F	CTTCACAGTTGGGTGGGAC	338 bp
gB-RT-R	GAGCCAGGGATTTGGATAG	
MDV-Meq-F	AGAGATGTCTCAGGAGCCAGAGCC	1020 bp
MDV-Meq-R	ATCATCAGGGTCTCCCGTCACCTG	
MDV-VP22-F	ATCGGATCCATGGGGGATTCTGAAAG	750 bp
MDV-VP22-R	ACACTCGAGTTATTCGCTATCACTGC

**Table 3:** GenBank accession numbers of the Meq amino acid sequences used in this study

Isolate	Country of Origin	Accession Number	Pathotype
Md5	USA	AAG14255.1	vvMDV
Md11	USA	AAS01627.1	vvMDV
GA	USA	AAF66798.1	vMDV
RB1B	USA	AY243332	vvMDV
RL	USA	AAR13332.1	vv + MDV
TK	USA	AAR13333.1	vv + MDV
N	USA	AAR13330.1	vv + MDV
New	USA	AAR13331.1	vv + MDV
U	USA	AAR13334.1	vv + MDV
W	USA	AAR13335.1	vv + MDV
X	USA	AAR13336.1	vv + MDV
571	USA	AAR13322.1	vMDV
573	USA	AAR13323.1	vMDV
595	USA	AAR13327.1	vvMDV
617A	USA	AAR13324.1	vMDV
637	USA	AAR13325.1	vMDV
643P	USA	AAR13328.1	vvMDV
660-A	USA	AAR13338.1	vv + MDV
648A	USA	AAR13337.1	vv + MDV
686	USA	AAR13339.1	vv + MDV
CVI988/Rispens	Netherlands	AAP06940.1	attMDV
814	China	AAL99997.1	attMDV
J-1	China	AEA06596.1	N.A.
LQQHR	China	AEM63533.1	N.A.
LSY2	China	AEM63534.1	N.A.
LMS	China	AEZ51745.1	vvMDV
GX0101	China	AFX97850.1	vvMDV
GX070060	China	ACA13267.1	vvMDV
GX070079	China	ACA13268.1	N.A.
BY	China/Tibet	AEA06593.1	N.A.
GXY2	China	ABQ23868.1	N.A.
LDH	China	AEM63523.1	N.A.
LYC	China	AEM63536.1	N.A.
YLO40920	China	ABA54944.1	N.A.
G2	China	AAM00003.1	vvMDV

Abbreviation: *Att* attenuated, *N.A* not available, *v* virulent, *vv* very virulent, *vv +* very virulent plus

**Table 4:** GenBank accession numbers of the L-meq amino acid sequences used in this study

Isolate	Country of Origin	Accession Number	Pathotype
CVI988/Rispens	Netherlands	ABF72204.1	attMDV
814	China	ADA83412.1	attMDV
GX060167	China	ACD75766.1	N.A.
HNGS201	China	CCO02736.1	N.A.
MDCC-MSB1	Japan	BAC57989.1	N.A.
MDCC-RP1	Japan	BAC57991.1	N.A.
BC-1	USA	AAR13319.1	vMDV
GA	USA	AAC54628.1	vMDV
JM10	USA	AAP06937.1	vMDV
JM/102 W	USA	ABG22905.1	vMDV
RM-1	USA	ABG22996.1	attMDV
CU-2	USA	ACF94907.1	mMDV
3004	Russia	ABS84657.1	attMDV

Abbreviation: *att* attenuated; *m* mild, *N.A* not available, *v* virulent, *vv* very virulent, *vv +* very virulent plus

**Table 5:** GenBank accession numbers of the vp22 amino acid sequences used in this study

Isolate	Country of Origin	Accession Number	Pathotype
Md5	USA	YP-001033978.1	vvMDV
CVI988/Rispens	Netherlands	ABF72291.1	attMDV
GA	USA	AAF66784.1	vMDV
RB1B	USA	ABR13137.1	vvMDV
Md11	USA	AAS01692.1	vvMDV
648A	USA	AFM7426.1	vv + MDV
549A	USA	ABV31181.1	vvMDV
571	USA	ABV31182.1	vMDV
584A	USA	ABV31183.1	vv + MDV
595	USA	ABV31184.1	vvMDV
686	USA	ABV31179.1	vv + MDV
JW/102 W	USA	ABV31188.1	vMDV
CU-2	USA	ABV31189.1	mMDV
R2/23	USA	ABV31191.1	N.A.
RM-1	USA	ABV31180.2	N.A.
CC/1409	China	AQN78018.1	N.A.
HS/1412	China	AQN78190.1	N.A.
J-1	China	AQN77146.1	N.A.
814	China	AEV55030.1	attMDV
GX0101	China	AFX97970.1	vvMDV
LMS	China	AEZ51712.1	vvMDV
JL/1404	China	AQN77845.1	N.A.
LCC	China	AQN77320.1	N.A.
LCY	China	ARE59101.1	N.A.
LTS	China	AQN77496.1	N.A.

Abbreviation: *att* attenuated, *N.A* not available, *v* virulent, *vv* very virulent, *vv +* very virulent plus

**Fig. 6:**
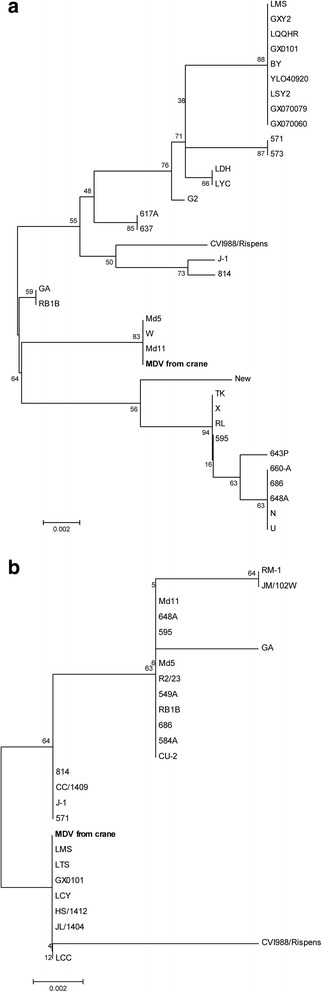
Phylogenetic profile showing the relationships among MDV isolates based on a comparison of the (**a**) Meq gene and (**b**) VP22 gene. Phylogenetic analysis of MDV based on (**a**) Meq and (**b**) VP22 amino acid sequences. The tree was constructed using the neighbor-joining (N-J) analysis method in the MEGA 5.0 program with bootstrapping (1000)

Phylogenetic analysis of the Meq gene showed that our MDV clustered with very virulent strain and was most closely related to Md5, Md11 and W (Fig. 6a). However, phylogenetic analysis of the VP22 gene showed that our MDV was closely related with attenuated virulent strain CVI988 (Fig. 6b).

**Fig. 7:**
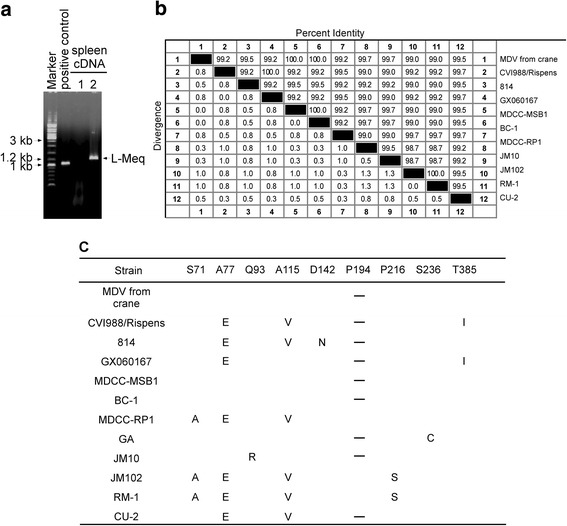
Amino acid sequence alignment of L-Meq (from crane). **a**, The full-length L-Meq gene (1197 bp) was amplified from spleen cDNA. **b**, The amino acid sequence of L-Meq (398 aa) from cranes was aligned with 11 previously published MDV isolates (including CVI988, 814, GX060167, MDCC-RP1, MDCC-MSB1, BC-1, GA, JM10, JM102, RM-1 and CU-2). Sequences were aligned using the DNAMAN software. **c**, Substituted amino acids are listed, while deleted amino acids are denoted by strips in the alignment

The Meq gene is only present in MDV1m and its gene product, MEQ, which is an MDV1-specific 339 amino acids protein with an N-terminal basic leucine zipper (bZIP) [27, 28]. L-Meq is a longer isoform of Meq that contains a 180 bp nucleotide insertion in the transactivation domain of Meq and is normally detected in non-oncogenic MDV1 vaccine strains such as CVI988 [29]. Interestingly, L-Meq was also amplified from crane spleen cDNA (Fig. 7a). Sequence alignment indicated that it shared 100% identity with the BC-1 strain (Fig. 7b). One substitution was found in the American strains GA and JM10 (Fig. 7c), and four substitutions were found in the JM102 strain (Fig. 7c).

## Discussion and conclusions

This article describes the first reported case of MD in red-crowned cranes and confirms that red-crowned cranes are susceptible to MDV infection. Previous research has suggested that multiple avian species can be infected by MDV such as Galliformes (turkeys [8], quails [9] and pheasants [10]), Strigiformes (owls), Anseriformes (ducks, geese, swans), and Falconiformes (kestrels). Therefore, this is also the first molecular detection of Marek’s disease virus in the order Gruiformes. One possible sources of infection for red-crowned cranes might be from migrating wild birds. Murata et al. tested feather-tip samples of wild geese from Japan and the Far East region of Russia by nested PCR, and 30% of analyzed white-fronted geese contained MDV [30], which suggested that migratory birds such as white-fronted geese could be MDV carriers during their migration. Previous research showed that MDV can be transmitted horizontally and across-species. Kenzy and Cho reported that a MDV-positive quail can transmit MD to monitor chicks by contact exposure [31]. Additionally, MDV is fairly resistant to environmental factors. Indeed, MDV can survive at least a few months in the dust at room temperature [32]. The infection source is also diverse, including chicken feather follicle, scurf, house dust, feces, and saliva. Many chickens with a normal appearance can be MDV carriers and spread infection. All of these provide a conducive environment for the spreading of MDV. Considering that the red-crowned cranes were fed in mesh cages that were accessible to wild birds and were never immunized with MDV vaccine before, it is possible that the MDV infection originated from migratory birds.

This case report describes the first detection and characterization of MDV from red-crowned cranes in a Chinese zoo. The molecular analysis of MDV genes suggested that the MDV (from crane) belongs to MDV serotype 1. The red-crowned cranes described in this study showed clinical signs of disease including decreased feed consumption, depression, paralysis, joint swelling, and eventual death. The results of necropsies of dead cranes revealed diffuse nodules in skin, muscle, liver, gizzard and heart. Histological investigation demonstrated lymphomatous infiltration into the affected tissue and neoplastic changes with evidence of mitosis. Additionally, a range of leukocytes was observed, including small/large lymphocytes, lymphoblasts, and occasional plasma cells. The demonstration of polymorphic leukocytes along with molecular detection is highly suggestive of Marek’s disease. RT-PCR targeting oncogenic gene Meq and glycoprotein B (gB) was performed to confirm the presence of MDV, with observation of expected amplification products throughout all tissue samples. Further phylogenetic analysis of the Meq and VP22 genes suggested that our MDV could belong to MDV-1, but it is a novel MDV that contains both Meq and L-Meq gene in its genome.

The detection of MDV in red-crowned cranes suggests that the virus may have a huge impact on the endangered crane’s survival in the wild. However, because this is the first verified instance of crane mortality resulting from MD, its true impact on the population is unknown. A possible solution for management of this species is to vaccinate all the red-crowed cranes to provide immunity against this disease. There are several available MDV vaccines including serotypes MDV-2 (SB-1), HVT, and attenuated MDV-1 (CVI988/Rispens), which are used singly or jointly [33]. MDV vaccination began in the late 1960s and made great contributions to protecting the poultry industry for more than 50 years. However, the current problem involves attenuated MDV vaccine that cannot provide effective protection against new virulent MDV strains. For example, previous reports have shown that molecular evolution of MDV genes could increase the pathogenicity of MDV virulent strains [34]. Infection of very virulent MDV (vvMDV) could also break post-vaccinal protection and cause the outbreak of MDV [35]. Therefore, the safety of this measurement needs to be studied in depth.

## Abbreviations

gB: Glycoprotein B
MDV: Marek’s disease virus
PCR: Polymerase chain reaction
